# Clinical Characteristics and Outcomes in Elderly Patients With COVID-19: A Single-Centre Retrospective Study

**DOI:** 10.7759/cureus.25506

**Published:** 2022-05-30

**Authors:** Kartik Mittal, Minakshi Dhar, Monika Pathania, Vartika Saxena

**Affiliations:** 1 Internal Medicine, All India Institute of Medical Sciences, Rishikesh, IND; 2 Community and Family Medicine, All India Institute of Medical Sciences, Rishikesh, IND

**Keywords:** elderly covid-19 patients, covid-19 india, critically ill elderly patients, covid-19 impact, outcomes in hospitalized covid-19 patients, hypertension and covid-19, covid 19

## Abstract

Background: Even with the wide base of knowledge that has been accumulated regarding coronavirus disease 2019 (COVID-19), only limited studies have tried to establish differences in outcomes of elderly patients hospitalized with COVID-19. We, thus, conducted a retrospective study on a large cohort of hospitalized patients with COVID-19 to improve the understanding of such differences and add to the evidence available regarding this age group.

Methods: This is a single-centre retrospective study conducted at a tertiary level hospital in the state of Uttarakhand in North India to determine clinical characteristics and outcomes in elderly patients (≥ 60 years) hospitalized with COVID-19 between May 1, 2020, and May 31, 2021. Our study included a retrospective follow-up at six months to also determine rehospitalizations and post-discharge mortality.

Results: There was a statistically significant difference (p<0.05) in in-hospital mortality, various in-hospital complications, duration of stay, number of rehospitalizations at six months, and post-discharge mortality up to six months in the elderly age group hospitalized with COVID-19.

Conclusions: This retrospective study demonstrates that the clinical characteristics and outcomes in hospitalized elderly with COVID-19 differ significantly from the younger adult population and demonstrates a need for greater hospital resource utilization in this age group. These results will help policymakers be better prepared for future pandemics.

## Introduction

Coronavirus is a heterogenous cluster of large single-stranded RNA viruses with alpha and beta genera that cause human infection. The common clinical presentation of this illness includes fever, cough, dyspnea, fatigue, myalgias, headache, loss of taste and smell, and can present as a severe illness, which includes extensive pulmonary involvement with acute respiratory distress syndrome (ARDS), multiple organ dysfunction syndrome (MODS), pulmonary thromboembolism, and cytokine storm, which lead to critical illness and in many cases, death [1,2]. It’s evident through common clinical experience that elderly patients with any infections may present atypically, which makes diagnosis difficult and delays management, thus increasing morbidity and mortality in this group of patients [3]. As comorbidities often increase with ageing, managing the illness becomes even more complicated. In fact, ageing itself is strongly associated with worse outcomes in any respiratory ailment, because of age-related weakening of respiratory anatomy and physiology, and diminishing pulmonary functional reserve [3,4]. Even age >80 years itself has shown to independently increase the risk of death in this subgroup in comparison to younger adults in any kind of lower respiratory infection [3,5]. Based on early data from China, case fatality rate (CFR) in patients over 60 years is higher than the mean CFR, with age-adjusted CFR being around 3.6% in the 60-69 year group, 8% in the 70-79 year group, and around 14.8% in the >80 years age group [4,6]. This subgroup, thus, also has increased utilization of intensive care and mechanical ventilation. CFR in coronavirus disease 2019 (COVID-19)-related ARDS has been seen to be 30-40% but can reach up to 70% in elderly patients. Studies also show a higher vulnerability of elderly men compared to women [7-9]. Similar patterns of severity and mortality have also been observed previously in other viral pneumonia [1].

The main reason for such differences in susceptibility and severity in pathogenesis is the age-related immune system remodelling, also called immunosenescence, which has previously widely been studied in other prevalent seasonal viral pneumonia-like influenza, and this concept extends to impaired immune responses to vaccination in elderly individuals [3,5,10]. Certain proteins of this virus have the ability to inhibit interferon response, specifically type 1, which in turn develops a protective mechanism that blocks further activation of CD8 cytotoxic T cells that now can’t kill this virus. This ability of the virus, when combined with the already weak interferon response of the elderly immune system, acts to double down to make the ageing population extremely susceptible to invasion as well as the progression of the disease. This becomes even more detrimental in those elderly with multiple comorbidities, each of which adds up to weaken the immune system by similar mechanisms. Then comes the newly understood role of latent infections that become increasingly active as age-related immune dysregulation increases, as has been observed in cases of persistent CMV infection [3,5,10]. The role of the inflammatory cascade, development of cytokine storm, and widespread prothrombotic response is well documented with this virus, both clinically and intricate molecular mechanisms that drive them [5,11]. Our institute was converted to a COVID-19 Care Center to cater to the population of Uttarakhand and its neighbouring states. We planned this study to identify the clinical characteristics and outcomes of elderly patients who were admitted to our centre.

## Materials and methods

**Figure 1:**
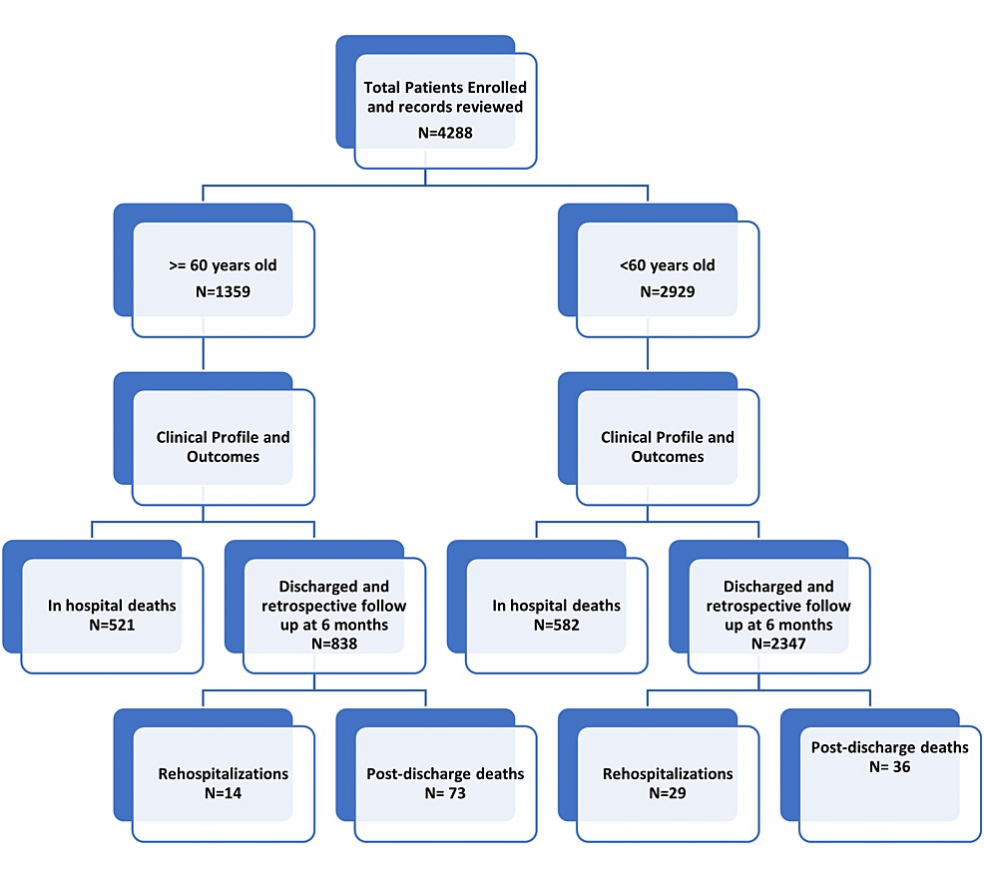
Study Flowchart

The present study was a single-hospital, retrospective observational study. The study was done by analyzing data of hospitalized COVID-19 patients in the All India Institute of Medical Sciences (AIIMS), Rishikesh, a tertiary level referral centre in Dehradun district of the mountain state of Uttarakhand in North India. It receives patients from its own state as well as the neighbouring densely populated states of Uttar Pradesh, Haryana, and Punjab. The study flow chart is shown in Figure 1. The study was done by utilizing data from May 1, 2020, to May 31, 2021, from all the available data records, both electronic and other available documents, and analyzing them retrospectively. The patient data were reviewed after clearance from Institutional Ethics Committee, via the Medical Records Department, of our institute. Electronically available records were assessed using the E-Hospital online portal, which is maintained by the Central Government of India as a storehouse of all digital health records of registered patients. All patients ≥18 years presenting with COVID-19 illness during the specified duration of the study were included. Records of patients with severe acute respiratory syndrome coronavirus 2 (SARS-CoV-2) reverse transcription-polymerase chain reaction (RT-PCR) positive reports or rapid antigen test (RAT) positive reports were enrolled for the study. Preformed performa was used to collect the data related to demographic and clinical characteristics including signs and symptoms of presentation and comorbidities. Data regarding level of care, which was defined as those receiving care in wards or in COVID-19 ICUs, were collected. Successful non-invasive ventilation (NIV) was defined as those patients who could be downgraded from NIV support to oxygen or the room air and failed NIV was defined as those patients who had to be upgraded to an invasive mechanical ventilator. Discharged patients were followed telephonically for three months for mortality. Patient data were divided into two groups: patients who were < 60 years were labelled as adults and those with age ≥ 60 years were labelled as elderly. 

Statistical analysis

The data were tabulated in an Excel Sheet (Microsoft Corporation, Redmond, Washington, United States), accordingly coded, and analyzed with IBM SPSS Statistics for Windows, Version 23.0 (Released 2015; IBM Corp., Armonk, New York, United States). Categorical variables were presented as numbers and percentages, and continuous or discrete variables as median, and mean with standard deviation (SD). Statistical differences were evaluated by Pearson’s chi-square or Fisher’s exact tests for categorical variables, as appropriate. Means of quantitative data were compared using paired t-test. The statistical significance was defined as p-value <0.05 and taking confidence level as 95%. The data underwent extensive descriptive and comparative analysis, along with multivariate regression analysis to determine the correlation between the most important variables and outcome.

This study was initiated only after obtaining clearance from the Institutional Ethics Committee, All India Institute of Medical Sciences, Rishikesh, Uttarakhand, India (AIIMS/IEC/21/428).

## Results

A total of 4288 patients were studied during the mentioned study duration. Among the study participants, 2929 (68.3%) were < 60 years (adult group) and 1359 (31.7%) were ≥ 60 years (elderly group). The mean age in the elderly group was 68.5 ± 6.9 years, and in the adult group was 41.5 ± 11.6 years. Out of the total participants, 2841 (66%) were males and 1447 (34 %) were females. Male predominance was also seen among the two groups; 66.0% males versus 34.0% females in the adult group and 66.8% males versus 33.2% females in the elderly group. Smoking was more prevalent in the elderly age group, 16% in the elderly versus 8.6% in the adult group.

Table 1 shows the overall distribution of comorbidity profile of the entire study population hospitalised with COVID-19, and distributed into age groups, with comorbidities most prevalent in the elderly group given in decreasing order as follows: hypertension, diabetes, heart disease, chronic kidney disease (CKD), chronic obstructive pulmonary disease (COPD), stroke, cancer, and chronic liver disease (CLD). In the adult group, diabetes was as prevalent as hypertension, followed by COPD, equal prevalence of CKD and heart disease, followed by CLD, cancer, and the least prevalent being stroke. 

**Table 1:** Comparison of comorbidities among hospitalized elderly and adult patients with COVID-19 COPD: Chronic Obstructive Airway Disease; CKD: Chronic Kidney Disease; CLD: Chronic Liver Disease; COVID-19: Coronavirus Disease 2019

Parameters	Age		p-value
	<60Years (n = 2929)	≥60Years (n = 1359)	
Co-morbidities			
Diabetes	560 (19.1%)	458 (33.7%)	<0.001
Hypertension	558 (19.1%)	679 (50.0%)	<0.001
Heart Disease	96 (3.3%)	236 (17.4%)	<0.001
COPD	104 (3.6%)	135 (9.9%)	<0.001
CKD	98 (3.3%)	200 (14.7%)	<0.001
CLD	86 (2.9%)	21 (1.5%)	0.007
Cancer	58 (2.0%)	27 (2.0%)	0.989
Stroke	50 (1.7%)	71 (5.2%)	<0.001
No comorbidities	1496 (51%)	133 (9.7%)	<0.001

Table 2 shows that fever was a predominant symptom in the adult group while cough was a predominant symptom in the elderly group. Shortness of breath was more prevalent in the elderly group (31% in the adult group versus 67.7% in the elderly group). Table 3 reveals that asymptomatic patients were more in the adult group (10.4% in the adult group versus 3.1% in the elderly group) and severe COVID-19 patients were more in the elderly group (30.4% in the adult group versus 52.9% in the elderly group).

**Table 2:** Comparison of symptoms at admission among hospitalized elderly and adult patients with COVID-19 SOB: Shortness of Breath; GI: Gastrointestinal; COVID-19: Coronavirus Disease 2019

Parameters	Age		p-value
	<60Years (n = 2929)	≥60Years (n = 1359)	
Symptoms at Admission			
Fever	2770 (94.6%)	1046 (77.0%)	<0.001
Cough	1839 (62.8%)	1135 (83.5%)	<0.001
Fatigue	1226 (41.9%)	626 (46.1%)	0.010
Headache	812 (27.7%)	263 (19.4%)	<0.001
Sore Throat	772 (26.4%)	249 (18.3%)	<0.001
Myalgia	782 (26.7%)	256 (18.8%)	<0.001
SOB	926 (31.6%)	920 (67.7%)	<0.001
GI Symptoms	194 (6.6%)	95 (7.0%)	0.656
Nasal/Conjunctival Congestion	28 (1.0%)	45 (3.3%)	<0.001

**Table 3:** Comparison of COVID-19 severity among hospitalized elderly and adult patients COVID-19: Coronavirus Disease 2019

Parameters	Age		p-value
	<60years (n = 2929)	≥60Years (n = 1359)	
COVID-19 Severity			
Asymptomatic	305 (10.4%)	42 (3.1%)	0.006
Mild	773 (26.4%)	177 (13.0%)	<0.001
Moderate	962 (32.8%)	421 (31.0%)	<0.001
Severe	889 (30.4%)	719 (52.9%)	<0.001

For analysis of the level of care, patients were divided into ward care and critical care groups as shown in Table 4. Those who did not need oxygen support and those requiring only non-invasive oxygen were kept in a ward-based setup, and those requiring NIV or mechanical ventilation were being managed in COVID-19 ICU. In the elderly group, a majority of patients needed ICU setup, while in the adult group, a majority were managed in the ward alone, without the need for critical care support. Patients in the adult group had a higher percentage of NIV success.

**Table 4:** Comparison of type of ventilatory support among hospitalized elderly and adult patients with COVID-19 NIV: Non-Invasive Ventilation; MV: Mechanical Ventilation; COVID-19: Coronavirus Disease 2019

Parameters	Age		p-value
	<60Years (n = 2929)	≥60Years (n = 1359)	
Type of Ventilatory Support			
None/Ward-Based (ward care)	2038 (69.6%)	640 (47.1%)	
1) None	1132 (38.6%)	241 (17.7%)	<0.001
2) O2	906 (30.9%)	399 (29.4%)	<0.001
NIV/MV (critical care)	891 (30.4%)	719 (52.9%)	
1) NIV	337 (11.5%)	221 (16.3%)	<0.001
2) MV	554 (18.9%)	498 (36.6%)	0.003
NIV Outcome- Success	337 (47.1%)	221 (38.2%)	<0.001

**Figure 2:**
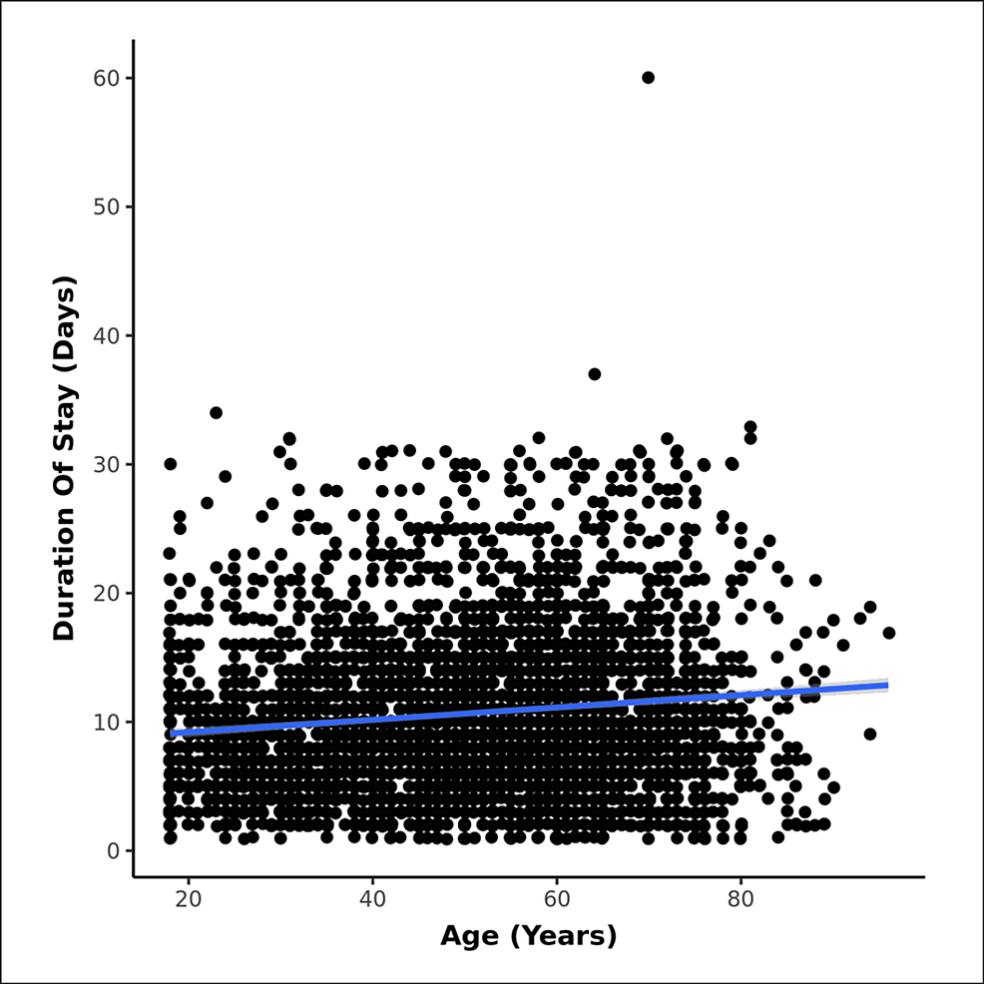
Correlation between age (years) and duration of stay (days): Scatter plot shows increasing duration of stay as the age of the hospitalised patients increases, with a weak positive correlation between age (years) and duration of stay (days), and this correlation was statistically significant (rho = 0.12, p = <0.001). For every 1 unit increase in age (years), the duration of stay (days) increased by 0.05 unit

Table 5 shows various in-hospital complications and their percentage distribution in both groups. Patients in the elderly group most commonly developed ventilator-associated pneumonia/hospital-acquired pneumonia (VAP/HAP), followed by ARDS and acute kidney injury (AKI), while those in the adult group most commonly developed ARDS. There was a higher prevalence of shock in the elderly group. Table 6 shows a comparison of various outcomes distributed according to age group, showing a longer mean duration of stay, and a higher rate of in-hospital and post-discharge mortality for the elderly group. Figure 2 shows the correlation between age and duration of hospital stay.

**Table 5:** Comparison of in-hospital complications among hospitalized elderly and adult patients with COVID-19 HAP: Hospital-Acquired Pneumonia; VAP: Ventilator Acquired Pneumonia; ARDS: Acute Respiratory Distress Syndrome; AKI: Acute Kidney Injury; PTE: Pulmonary Thromboembolism; COVID-19: Coronavirus Disease 2019

Parameters	Age		p-value
	<60Years (n = 2929)	≥60Years (n = 1359)	
In-Hospital Complications			
HAP/VAP	534 (18.2%)	439 (32.3%)	<0.001
ARDS	802 (27.4%)	585 (43.0%)	<0.001
AKI	173 (5.9%)	374 (27.5%)	<0.001
Shock	175 (6.0%)	179 (13.2%)	<0.001
PTE	135 (4.6%)	42 (3.1%)	0.020
Mucormycosis	55 (1.9%)	19 (1.4%)	0.262

**Table 6:** Comparison of duration of stay and outcomes among hospitalized elderly and adult patients with COVID-19 COVID-19: Coronavirus Disease 2019

Parameters	Age		p-value	
	<60Years (n = 2929)	≥60Years (n = 1359)		
Comparison of Outcomes				
Duration Of Stay (Days)	10.25 ± 5.88	11.50 ± 6.81	<0.001	
In-Hospital Mortality	582 (19.9%)	521 (38.3%)	<0.001	
Post-Discharge Mortality	36 (1.6%)	73 (13.4%)	<0.001	
Overall Mortality	618 (21.1%)	594 (43.7%)	<0.001	

## Discussion

This was a descriptive study to find out the differences in clinical characteristics of elderly patients and adults hospitalized with COVID 19. The mean age in the elderly group was 68.5± 6.9 years, and the adult group was 41.5 ± 11.6 years. In both elderly and adult groups, around two-thirds of admitted patients were males. This inequality in gender affliction of hospitalized patients with COVID-19 correlated with a study of characteristics by Guan et al. in China [2]. In terms of risk factor comparison, 16% of elderly patients were smokers, compared to 8.6% in adults less than 60 years, showing a higher prevalence of smoking in elderly admitted with COVID-19, which was statistically significant (<0.001). 

Our detailed analysis showed the comparison of comorbidities in both age groups. The distribution of comorbidity in these hospitalized patients with COVID-19 showed a statistically significant burden of comorbidities in the elderly group. Among the elderly age group, around half of the admitted patients were hypertensive, and one third were diabetic. Other comorbidities Like heart disease (17%), CKD (15%), and COPD (10%) were also common in this group. On the other hand, in those younger than 60 years, the adult group, one-fifth of them were either hypertensive or diabetic. Comorbidity profile of our study correlated well with the study of hospitalized COVID-19 patients by Wei et al. [12] in China, owing to quite a similar rate of comorbidity prevalence in both the South Asian countries, except for diabetes, which was twice as prevalent in our Indian study compared to theirs. Our study was also comparable to other characteristic studies describing comorbidities [7,12-14].

A comparison of symptomatology at admission in both groups showed that among the elderly, cough (84% vs 63%) and shortness of breath (68% vs 32%) were more common, while fever (77% vs 95%), headache (19% vs 28%), sore throat (18% vs 26%) and myalgia (19% vs 27%) were much less common, all of which showed statistically significant distribution (p<0.001). GI symptoms were uncommon on admission (7% vs 6.6%, p=0.656), and were comparable in both age groups. These results correlate well with a Chinese study by Guo et al. [15], in which the prevalence of cough in the elderly was 80%, and a study by Wei et al. [12], where the prevalence of fever in the elderly was 80%. However, the prevalence of dyspnoea varied widely in studies, where the prevalence of shortness of breath in our study compared to Guo et al. [15] and Wei et al. [12] was around 70%, 40%, and 15%, respectively.

We also analyzed the vitals of COVID-19 patients on admission to the Emergency Department to see the differences in the response of the elderly patient’s body to COVID-19 infection like any other viral infection. Compared to younger adults, elderly patients showed higher mean heart rate (123 ± 16 vs 120 ± 12, p<0.001), systolic blood pressure (123 ± 28 vs 117 ± 18, p<0.001), diastolic blood pressure (77 ± 16 vs 76 ± 12, p<0.001), temperature (101.9 ± 2.9 vs 101.5 ± 1.3, p<0.001), and lower admission SpO2 (88 ± 4 vs 89 ± 3, p<0.001), which shows more effect of this virus on heart rate, temperature, and SpO2 in the elderly than in adults younger than 60 years, and the higher distribution of hypertension in the elderly owing to the higher mean pressures. Mean standard deviations were higher for older adults in all vital parameters. These results were comparable to studies by Wei et al. [12] and Guo et al. [15].

In our data, it was important to compare the distribution of COVID-19 severities in both age groups, and the elderly group had a disproportionately higher proportion of patients who were admitted with severe disease (53% vs 30%, p<0.001). One-third of patients in both groups had a moderate disease (refer to Table 5). Our study had more prevalence of severe disease as compared to other studies, which might be due to the nature of the referral system in our setup, the sickest of patients are referred to our tertiary level centre, which caters to a high burden of critically ill patients.

It had become imperative with this pandemic that the level of healthcare utilization must be judicious, and owing to the paucity of resources in a developing country like India, we were bound to study the differences in resource utilization by these two age groups, to make us better prepared in future to be more rational in resource distribution, and guide policymakers to be ready to allocate more funding to healthcare needs of the elderly, the age group bound to increase exponentially in the coming years. We analyzed the utilization of resources using the need for a non-O2 bed, oxygen bed, non-invasive mask ventilation, and mechanical ventilation. As per our institutional policy, oxygen-requiring patients were managed in a ward-based setting, while those requiring mechanical ventilation including NIV or invasive ventilation were shifted to, or directly admitted from the Emergency Department, to a critical care unit. During this pandemic, our centre was declared a COVID-19 hospital, and resources were ramped up to expand our ICU capacity. The requirement for any type of mechanical ventilatory support (non-invasive or invasive) was statistically more significant in elderly patients than in adults less than 60 years (refer to Table 6). Thus, the occupancy of the critical care unit was higher for the elderly group. The odds ratio for the need for critical care support in the elderly group compared to the adult group was 2.57 (95% CI, 2.25-2.93, p<0.001). This correlated with the Italian study by Dres et al. [16], which showed higher utilization of ICU resources by older adults. In another Italian study by Boscolo et al. [17], elderly patients (older than 73 years) showed higher in-hospital mortality than younger adults. This shows that we need to be ready for future pandemics with higher resources for the rising aging population to prevent pressure on our health systems.

To highlight the higher risk of in-hospital complications in elderly patients, we analyzed the differences in the most commonly encountered complications in both age groups. While most prevalent in-hospital complication in both groups was ARDS, however, ARDS (43% vs 27%, p<0.001), HAP/VAP (32% vs 18%, p<0.001), AKI (28% vs 6%, p<0.001), and shock (13% vs 6%, p<0.001) were much more prevalent in the elderly group compared to the adult group. The odds ratio for development of respective in-hospital complications in the elderly group compared to the younger adults was 2.14 (95% CI, 1.85-2.48, p<0.001) for HAP/VAP, 2.0 (95% CI, 1.75-2.29, p<0.001) for ARDS, 6.05 (95% CI, 4.98-7.35, p<0.001) for AKI, and 2.39 (95% CI, 1.92-2.97, p<0.001) for shock. Our study had a greater prevalence of ARDS and AKI in the elderly compared to Guo et al. (30% ARDS, 15% AKI) and Wei et al. (29% ARDS, 4% AKI) and some other studies [12,15]. Studies by Povoa et al. and Maes et al. have shown a higher vulnerability of hospitalized COVID-19 patients to developing in-hospital pneumonia, which is strongly related to the duration of ventilation, presence of ARDS, proning duration, male gender, use of high dose immunosuppressants, and the causative organism, similar to other critically ill patients [18,19].

Analysis was also done to compare the differences in duration of stay between the two age groups and the mean stay for the elderly group was higher (11.5 ± 6.8 days vs 10.25 ± 5.8 days, p<0.001). For every one-year increase in age, the risk of increase in the duration of stay increased by 0.05 days. Our study showed the duration of stay comparable to the Chinese study by Guan et al. [5], where the mean duration of stay of overall data was 12.8 ± 2 days, and a German study by Gunster et al. where it was 14 ± 2 days [20]. Our study involved a follow-up period of six months, where we retrospectively analyzed the effect of various parameters on post-discharge death and rehospitalization by contacting the patients’ families telephonically only once six months after they were discharged. A total of 73 patients in the elderly group and 36 patients in the adult group had died on follow-up. The mean age of those who died six months post-discharge was 67±6 years in the elderly group, and 46±11 years in the adult group (p=0.004). As with in-hospital mortality data, post-discharge death analysis too showed a preponderance of male gender in both age groups (refer to Table 19). The odds ratio for post-discharge mortality in the elderly group was 9.43 (95% CI, 6.25-14.23, p<0.001). We compared our results with those of the study by Gunster et al., who retrospectively analyzed their discharged patients for up to six months period. The mean age of their analysis was 79±13 years [20].

There are a few limitations of this study which we have identified. First, this is a retrospective study, and more prospective studies in future might be more significant in terms of evidence. Second, this is a single-centre study, which makes the population analysed in our study quite homogenous. Also, this study spanned multiple waves of the pandemic in India, and time-based precise analysis of these trends was not possible in the way data was collected. Lastly, our study did not include the effects of experimental therapies and vaccines on outcomes of hospitalized patients. 

## Conclusions

In this study, we showed how elderly patients were disproportionately affected by the COVID-19 pandemic in terms of both in-hospital and long-term outcomes. These results will guide policymakers to be better prepared for the future, where the representation of this age group in the overall population is bound to increase exponentially.
